# Prostatic Carcinosarcoma with Lung Metastases

**DOI:** 10.1155/2013/790790

**Published:** 2013-11-05

**Authors:** Stefanie R. Furlan, David J. Kang, Armando Armas

**Affiliations:** ^1^Nova Southeastern University College of Osteopathic Medicine, 3301 College Avenue, Fort Lauderdale, FL 33314, USA; ^2^Calaway Young Cancer Center, Valley View Hospital, 1906 Blake Avenue, Glenwood Springs, CO 81601, USA

## Abstract

Carcinosarcoma of the prostate is an uncommon malignancy with poor long-term prognosis. The cancer is typically discovered at an advanced stage, and with less than 100 reported cases, there is limited literature concerning treatment options. Our patient presented with a history of benign prostatic hypertrophy, erectile dysfunction, and nocturia. Biopsy of his prostate indicated that the patient had prostatic adenocarcinoma, but histopathology after prostatectomy revealed carcinosarcoma. It has been over six years since this patient's diagnosis of carcinosarcoma. Over this span of time, he has received a radical prostatectomy, radiotherapy, and androgen ablative therapy. The patient also developed multiple lung metastases that have been treated with video-assisted thoracic surgery and stereotactic body radiosurgery. Overall, he has remained unimpaired and in good condition despite his aggressive form of cancer.

## 1. Introduction

Carcinosarcoma of the prostate is also known as sarcomatoid carcinoma [1–4]. This malignancy has a very poor prognosis, with a survival of approximately 7 months. Almost 25% of patients have metastatic disease at the time of diagnosis [1, 2]. Carcinosarcoma is a histological mixture of undifferentiated adenocarcinoma and varying types of sarcoma, including, but not limited to, osteosarcoma, chondrosarcoma, and fibrosarcoma [2]. In previous cases, no variables including patient's age, components of the sarcoma, or therapy were found to be statistically relevant in predicting overall outcome [2]. This case report highlights a patient who has done relatively well with metastatic prostatic carcinosarcoma for over 6 years, despite his poor prognosis.

## 2. Case Report

A 72-year-old male with a longstanding history of benign prostatic hypertrophy (BPH) and erectile dysfunction (ED) initially presented with severe nocturia that had been present for several years. On digital rectal exam, a diffusely enlarged prostate with discrete nodularity and central induration was palpated. Prostate-specific antigen level (PSA) was 2.9 ng/dL (<4 ng/mL). A subsequent biopsy revealed Gleason 8, T2 adenocarcinoma of the prostate.

**Figure 1:**
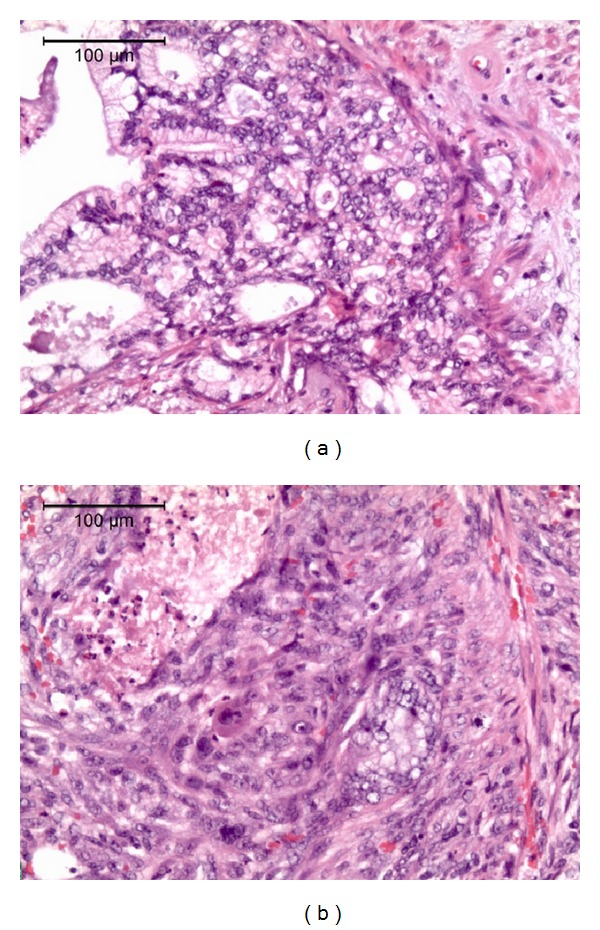
(a) Histologic section of prostatic carcinosarcoma with characteristic findings. (b) Histologic section of prostatic carcinosarcoma with characteristic findings.

**Figure 2:**
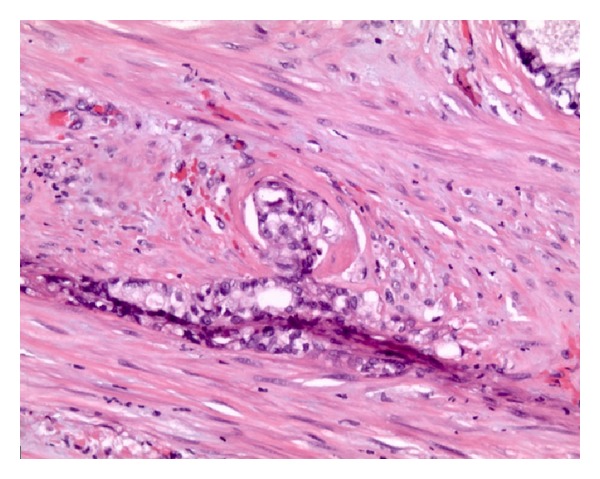
Histologic section of prostatic carcinosarcoma with lymphovascular and perineural invasion.

**Figure 3:**
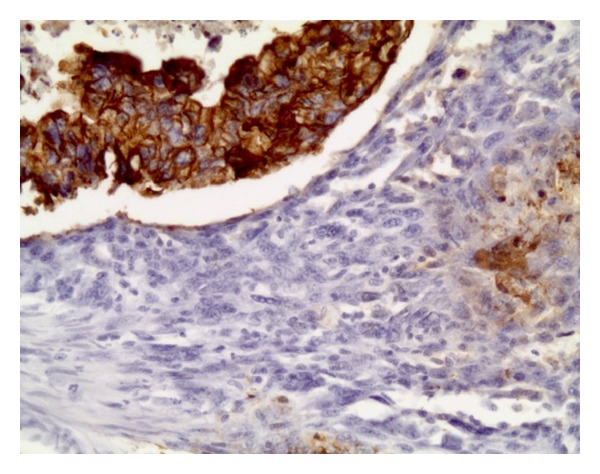
Immunohistochemical findings. CK7 positive tumor cells.

Three months after diagnosis of prostatic adenocarcinoma, the patient underwent a retropubic radical prostatectomy and bilateral pelvic lymph node dissection. The prostatic volume measured 75 cc. Contrary to the initial biopsy, the histopathology of the specimen indicated carcinosarcoma (Figures 1(a) and 1(b)) of the prostate with seminal vesicle, lymphovascular, and perineural invasion (Figure 2). The specimen was CK20 negative, CK7 positive (Figure 3), and PSA negative. The diagnosis of prostatic carcinosarcoma was confirmed by Johns Hopkins University, with a Gleason Grade (5 + 4). Metastatic workup was negative. Repeat PSA levels were <0.1 ng/dL or undetectable. The patient completed a course of pelvic radiotherapy following surgery and began androgen ablative therapy with leuprolide and triptorelin. Though the patient developed proctosigmoiditis secondary to radiotherapy, the condition resolved with hyperbaric oxygen therapy (HBO).

The patient had no evidence of disease (NED) for approximately 15 months, until followup PET/CT revealed a 1.5 cm left pulmonary nodule. The lesion was resected with video-assisted thoracic surgery (VATS). The pulmonary nodule was a circumscribed tumor composed of a proliferation of neoplastic glands with moderate differentiation and admixed stromal cells which had significant anaplasia and areas of tumor necrosis. The lesion was prostatic acid phosphatase positive, PSA negative, and CK7 positive, thus indicating metastatic disease of prostatic origin.

Following pulmonary wedge resection, the patient received an artificial urinary sphincter and male sling for urinary incontinence secondary to the initial prostatectomy. The patient remained clinically free of disease for another 9 months, until which a 3 mm nodule appeared in left upper lobe on chest CT. The nodule was treated with stereotactic body radiosurgery, at a dose of 5200 cGy in four fractions.

Three years later, five new lung metastases were described on CT. The highly suspicious spherical nodules involved both lungs and measured from 3 to 8 mm in diameter. No significant fludeoxyglucose (FDG) uptake was observed on PET/CT. Stereotactic body radiosurgery was performed on the two largest lesions, at a dose of 5200 cGy in four fractions. All lesions noticeably responded to treatment via the abscopal effect.

The most recent chest CT revealed a well-defined 6 mm nodule in the right middle lobe and a 4 mm paravertebral nodule in the right base. Scattered bilateral areas of patchy density suggestive of posttherapy change and scarring in left upper lobe secondary to VATS were also observed. At this time, the lesions are being monitored for any change. The patient has also discontinued androgen ablative therapy due to vasomotor side effects. PSA and serum testosterone are routinely monitored and remain low. Recent laboratory values described a PSA <0.1 ng/dL and serum testosterone of 97 ng/dL.

For over six years, the patient has continued to remain functional and asymptomatic despite a diagnosis of metastatic carcinosarcoma of the prostate. The combination of radical prostatectomy, radiotherapy, androgen ablative therapy, and stereotactic body radiosurgery has proven to be successful palliative treatment thus far.

## 3. Discussion

Carcinosarcoma of the prostate is a rare malignancy characterized by adenocarcinoma admixed with sarcoma; this intertwined mixture of malignant epithelial and mesenchymal components is a highly aggressive neoplasm. In some publications, carcinosarcoma may also be referred to as sarcomatoid carcinoma [1–4]. There are fewer than 100 reported cases described in the literature. Most cases of prostate cancer are adenocarcinomas; less than 0.1% of all prostate malignancies are described as sarcoma. The origin of prostatic carcinosarcoma is unknown; some have proposed that both carcinoma and sarcoma simultaneously develop within the prostatic tissues, while others have suggested that adenocarcinoma has undergone transformation into sarcoma [2, 3]. Interestingly, 50% of prostatic carcinosarcomas have developed in patients with a history of prostatic adenocarcinoma [1, 4]. Patients with carcinosarcoma often have a low serum PSA compared to those with adenocarcinoma. Sarcomatoid components present in carcinosarcomas may include osteosarcoma, chondrosarcoma, rhabdomyosarcoma, leiomyosarcoma, and malignant fibrous histiocytoma. The malignant neoplasm may also contain multiple sarcomatoid entities [3, 4]. Carcinosarcoma tumors may be composed of 5–99% sarcoma [5, 6]. The sarcomatous components receive a Gleason grade of 5, while the glandular components are graded according to the standardized Gleason grade system [7].

The mean age of diagnosis is 66 years old [2]. There is a 20% risk of death within the first year of diagnosis [5, 6]. Long-term survival is quite rare for carcinosarcoma. A case series of 21 patients reported that a 5-year survival is 41% and 7-year survival is 14%. Approximately 25% of patients have metastatic disease at the time of diagnosis. Metastases most commonly occur in the lung (43%), bone (26%), and lymph nodes (19%) secondary to hematogenous spread [1, 4]. Patients diagnosed with carcinosarcoma commonly present with vague, nondiagnostic symptoms, such as urinary hesitancy, incomplete voiding, and weak urinary stream. Less common symptoms may include urinary frequency, urgency, dysuria, hematuria, and/or perineal pain [5].

There has been relatively little correlation between treatment and survival. Reported treatment modalities include transurethral resection, orchiectomy, chemotherapy, radiation, androgen ablative therapy, prostatectomy, cystoprostatectomy, and pelvic exenteration [2]. Radical prostatectomy is considered one of the best treatment modalities for prostatic carcinosarcoma; techniques such as transurethral resection have a far greater risk of incomplete resection. Even with complete resection, the incidence of local recurrence and/or metastases is high compared to other prostate malignancies. Currently, there is no standardized treatment regimen for prostatic carcinosarcoma [8].
